# ^18^F-MK-6240 tau PET in patients at-risk for chronic traumatic encephalopathy

**DOI:** 10.1186/s13024-025-00808-1

**Published:** 2025-02-25

**Authors:** Michael L. Alosco, Jhony Mejía Pérez, Julia E. Culhane, Ranjani Shankar, Christopher J. Nowinski, Samantha Bureau, Nidhi Mundada, Karen Smith, Alinda Amuiri, Breton Asken, Jenna R. Groh, Annalise Miner, Erika Pettway, Sydney Mosaheb, Yorghos Tripodis, Charles Windon, Gustavo Mercier, Robert A. Stern, Lea T. Grinberg, David N. Soleimani-Meigooni, Bradley T. Christian, Tobey J. Betthauser, Thor D. Stein, Ann C. McKee, Chester A. Mathis, Eric E. Abrahamson, Milos D. Ikonomovic, Sterling C. Johnson, Jesse Mez, Renaud La Joie, Daniel Schonhaut, Gil D. Rabinovici

**Affiliations:** 1 Department of Neurology, Boston University Alzheimer’s Disease Research Center, Boston University CTE Center, Boston University, Chobanian & Avedisian School of Medicine, Boston, MA USA; 2 Department of Neurology, Boston Medical Center, Boston, MA USA; 3 Department of Anatomy & Neurobiology, Boston University Chobanian & Avedisian School of Medicine, Boston, MA USA; 4 Department of Neurology, Alzheimer’s Disease Research Center, Memory & Aging Center, University of California San Francisco, San Francisco, CA USA; 5 Concussion Legacy Foundation, Boston, MA USA; 6 Department of Clinical & Health Psychology, 1Florida Alzheimer’s Disease Research Center, Fixel Institute for Neurological Diseases, University of Florida, Gainesville, FL USA; 7 Department of Biostatistics, Boston University School of Public Health, Boston, MA USA; 8 Molecular Imaging and Nuclear Medicine, Boston Medical Center, Boston, MA USA; 9 Department of Neurosurgery, Boston University, Chobanian & Avedisian School of Medicine, Boston, MA USA; 10 Department of Pathology, University of California San Francisco, San Francisco, CA USA; 11 Wisconsin Alzheimer’s Disease Research Center, School of Medicine and Public Health, University of Wisconsin-Madison, Madison, USA; 12 Department of Medicine, University of Wisconsin-Madison School of Medicine and Public Health, Madison, WI USA; 13 Department of Medical Physics, University of Wisconsin-Madison School of Medicine and Public Health, Madison, WI USA; 14 U.S.Department of Veteran Affairs, VA Boston Healthcare System, Jamaica Plain, MA USA; 15 Department of Psychiatry and Ophthalmology, Boston University Chobanian & Avedisian School of Medicine, Boston, MA USA; 16 Department of Pathology and Laboratory Medicine, Boston University Chobanian & Avedisian School of Medicine, Boston, MA USA; 17 Department of Radiology, University of Pittsburgh School of Medicine, Pittsburgh, PA USA; 18 Department of Neurology, University of Pittsburgh School of Medicine, Pittsburgh, PA USA; 19 Geriatric Research Education and Clinical Center, VA Pittsburgh HS, Pittsburgh, PA USA; 20 Department of Psychiatry, University of Pittsburgh School of Medicine, Pittsburgh, PA USA; 21 School of Medicine and Public Health, Wisconsin Alzheimer’s Institute, University of Wisconsin-Madison, Madison, USA; 22 Department of Radiology & Biomedical Imaging, University of California San Francisco, San Francisco, CA USA; 23 University of California, San Francisco (UCSF), Memory and Aging Center MC: 1207, 675 Nelson Rising Lane, Suite 190, San Francisco, CA 94158 USA

**Keywords:** Alzheimer’s disease, Biomarkers, Chronic traumatic encephalopathy, MK-6240, Repetitive head impacts, Tau PET

## Abstract

**Background:**

Molecular biomarkers of chronic traumatic encephalopathy (CTE) are lacking. We evaluated ^18^F-MK-6240 tau PET as a biomarker for CTE. Two studies were done: (1) ^3^H-MK-6240 autoradiography and an *in-vitro* brain homogenate binding studies on postmortem CTE tissue, (2) an *in-vivo* ^18^F-MK-6240 tau PET study in former American football players.

**Methods:**

Autoradiography and in-vitro binding studies were done using ^3^H-MK-6240 on frozen temporal and frontal cortex tissue from six autopsy cases with stage III CTE compared to Alzheimer’s disease. Thirty male former National Football League (NFL) players with cognitive concerns (mean age = 58.9, SD = 7.8) completed tau (^18^F-MK-6240) and Aβ (^18^F-Florbetapir) PET. Controls included 39 Aβ-PET negative, cognitively normal males (mean age = 65.7, SD = 6.3). ^18^F-MK-6240 SUVr images were created using 70–90 min post-injection data with inferior cerebellar gray matter as the reference. We compared SUVr between players and controls using voxelwise and region-of-interest approaches. Correlations between ^18^F-MK-6240 SUVr and cognitive scores were tested.

**Results:**

All six CTE stage III cases had Braak NFT stage III but no neuritic plaques. Two had Thal Phase 1 for Aβ; one showed a laminar pattern of ^3^H-MK-6240 autoradiography binding in the superior temporal cortex and less so in the dorsolateral frontal cortex, corresponding to tau-immunoreactive lesions detected using the AT8 antibody (pSer202/pThr205 tau) in adjacent tissue sections. The other CTE cases had low frequencies of cortical tau-immunoreactive deposits and no well-defined autoradiography binding. In-vitro ^3^H-MK-6240 binding studies to CTE brain homogenates in the case with autoradiography signal indicated high binding affinity (K_D_ = 2.0 ± 0.9 nM, B_max_ = 97 ± 24 nM, *n* = 3). All NFL players had negative Aβ-PET. There was variable, low-to-intermediate intensity ^18^F-MK-6240 uptake across participants: 16 had no cortical signal, 7 had medial temporal lobe (MTL) uptake, 2 had frontal uptake, and 4 had MTL and frontal uptake. NFL players had higher SUVr in the entorhinal cortex (d = 0.86, *p* = 0.001), and the parahippocampal gyrus (d = 0.39, *p* = 0.08). Voxelwise regressions showed increased uptake in NFL players in two bilateral anterior MTL clusters (*p* < 0.05 FWE). Higher parahippocampal and frontal–temporal SUVrs correlated with worse memory (*r* = -0.38, *r* = -0.40) and semantic fluency (*r* = -0.38, *r* = -0.48), respectively.

**Conclusion:**

We present evidence of ^3^H-MK-6240 in-vitro binding to post-mortem CTE tissue homogenates and in vivo ^18^F-MK-6240 PET binding in the MTL among a subset of participants. Additional studies in larger samples and PET-to-autopsy correlations are required to further elucidate the potential of ^18^F-MK-6240 to detect tau pathology in CTE.

**Supplementary Information:**

The online version contains supplementary material available at 10.1186/s13024-025-00808-1.

## Background

Chronictraumatic encephalopathy (CTE) is a neurodegenerative disease caused in part by exposure to repetitive head impacts (RHI) through contact and collision sports and other sources [1–3]. CTE is characterized by the perivascular accumulation of hyper-phosphorylated tau (p-tau) in neurons at the depths of cortical sulci [4, 5]. Current CTE staging schemes propose that the p-tau neuropathology accumulates in the frontotemporal cortices in the early (i.e., low) stages of the disease (stage I/II), with involvement of the medial temporal lobes (MTL) in later (i.e., high) disease stages (stage III/IV) [5, 6]. In addition to differences in distribution, CTE p-tau lesions are unique in their molecular structure from tau aggregates in other neurodegenerative diseases, such as Alzheimer’s disease (AD) or aging-related tau astrogliopathy (ARTAG) [7–10]. Unlike AD, neuritic amyloid-beta (Aβ) plaques are not an early feature of CTE and tend to accumulate with older age and co-morbid disease [5, 11].

CTE can only be diagnosed by post-mortem neuropathological examination. There are currently no validated in vivo biomarkers to detect and support a diagnosis of CTE during life. Tau PET imaging is a useful biomarker for quantifying tau deposition in AD and has been examined in CTE [12–21]. To date, most research has focused on ^18^F-flortaucipir (FTP), a first generation radioligand that has high affinity to the paired helical filaments of tau in AD neurofibrillary tangles [22–25]. Among people at high risk for CTE (i.e., older former National Football League [NFL] players), FTP tracer binding patterns are consistent with the distribution of neuropathology of CTE. However, the effect sizes are small in differentiating people at high risk for CTE from control groups, there is low signal intensity, and there is inconsistency in the literature [17, 20, 21, 26, 27]. FTP-to-autopsy studies similarly show at best modest correlations with CTE p-tau pathology [14, 27].

^18^F-MK-6240 is a second generation tau radioligand that shows greater dynamic range in AD and less intracerebral off-target binding than FTP [15, 16, 23, 24, 28]. Compared to other tau tracers, ^18^F-MK-6240 has a distinct chemical structure that may lead to unique binding affinities. Unlike FTP, ^18^F-MK-6240 rarely exhibits off-target binding in the basal ganglia and choroid plexus, but it does exhibit tracer retention in neuromelanin- and melanin-containing cells and meninges [22, 23]. ^3^H-MK-6240 demonstrated negligible binding to tau aggregates in CTE cases at stages II/III to IV in an autoradiography study across multiple tauopathies [24, 29]. Other autoradiography studies found ^3^H-MK-6240 binding in tissue with severe CTE (stage IV) but it was in the presence of AD [30]. A case report of ^18^F-MK-6240 PET in a symptomatic retired Australian-rules football player did show that ^18^F-MK-6240 binding patterns were consistent with the expected neuroanatomical distribution of CTE p-tau pathology [31]. The footballer had a higher Standardized Uptake Value ratio (SUVr) for all brain regions when compared to healthy controls, and higher SUVr in the dorsolateral prefrontal cortex (DLPFC) compared to a mild cognitive impairment (MCI) cohort. However, interpretation of that finding is challenging since the patient also had positive Aβ PET, suggesting the presence of AD copathology.

Here, we investigated the usefulness of ^18^F-MK-6240 tau PET for the detection of CTE. First, we report on ^3^H-MK-6240 autoradiography and an in-vitro binding analysis in brain tissue from six brain donors with autopsy-confirmed CTE. Next, we evaluated ^18^F-MK-6240 tau PET in 30 middle aged to older adult cognitively symptomatic former NFL players, a population at high risk for having underlying CTE [1].^ 18^F-MK-6240 tracer binding was compared to sample of cognitively unimpaired males from the Boston University (BU) and University of California San Francisco (UCSF) AD Research Centers (ADRCs) and the Wisconsin Registry for Alzheimer’s Prevention (WRAP) study. Longitudinal changes in ^18^F-MK-6240 PET were evaluated in four former NFL players (13–18 months follow up). Among the former NFL players, we evaluated the association between ^18^F-MK-6240 binding and metrics of exposure to RHI (i.e., years of American football play, age of first exposure to football) and measures of cognitive function.

## Methods

### Part I: Post-mortem study: Autoradiography and in-vitro binding

Postmortem studies were approved by the University of Pittsburgh’s Committee for Oversight of Research and Clinical Training Involving Decedents. Frozen brain tissue samples of superior temporal cortex and dorsolateral frontal cortex used for the autoradiography and in-vitro binding studies were obtained from the UNITE Brain Bank at the Boston University (BU) CTE Center. The UNITE Brain Bank is described elsewhere [32]. Briefly, it is a brain repository devoted to the study of the long-term effects of exposure to RHI on the central nervous system. It is enriched for tissue with autopsy-confirmed CTE. All brain donors have a history of exposure to RHI and being symptomatic is not a requirement for brain donation. Brain donors with poor tissue quality are excluded. Neuropathological diagnoses are made blinded to clinical data. The processing and assessment methods are described elsewhere. Established criteria are used for the neuropathological diagnosis of AD [33, 34], Lewy body disease [35], and frontotemporal lobar degeneration [36–38]. Methods for the evaluation and diagnosis of TDP-43 inclusions and white matter and vascular pathologies (i.e., cerebral amyloid angiopathy, arteriolosclerosis, atherosclerosis) have been described previously [39, 40].

For this study, six cases with autopsy-confirmed CTE stage III (of IV) [6] were selected. CTE was neuropathologically diagnosed based on published criteria [4, 5]. The six autopsy-confirmed CTE cases selected were required to not have co-morbid neurodegenerative disease diagnoses (specifically other tauopathies) or coexisting neuritic plaque pathology (CERAD score 0). A “positive control” case was a typical AD (Alzheimer’s Disease Neuropathologic Change: A3, B3, C3) from the University of Pittsburgh Alzheimer’s Disease Research Center brain bank. The assessment of regional p-tau severities as well as CTE stage and other neuropathological diagnoses (Table 1) was made at BU using tissue sections from the fixed hemisphere. To obtain p-tau severity ratings within the frontal and temporal cortex areas that were sampled for autoradiography from the opposite (frozen) hemisphere, frequency ratings of AT8 immunoreactive lesions were determined in areas of their highest involvement in tissue sections immediately adjacent to those assessed for autoradiography.

**Table 1:** Characteristics of six male brain donors with autopsy-confirmed CTE Stage III

	**Case 1**	**Case 2**	**Case 3**	**Case 4**	**Case 5**	**Case 6**
Age	52	69	64	66	70	87
Primary Exposure	Football (20 yrs)	Football (21 yrs)	Football (8 yrs)	Football (19 yrs)	Ice hockey (25 yrs)	Football (17 yrs)
Alzheimer’s Disease Neuropathologic Change	Not AD	Not AD	Not AD	Not AD	Low	Low
Thal phase	0	0	0	0	1	1
Braak tau NFT stage	III	III	III	III	III	III
CERAD	0	0	0	0	0	0
Lewy body disease	Absent	Absent	Absent	Absent	Absent	Absent
TDP-43	Absent	Amygdala, Entorhinal	Absent	Absent	Amygdala, Entorhinal, Frontal	Amygdala, Hippocampus Entorhinal
Cerebral amyloid angiopathy	Absent	Absent	Absent	Absent	Absent	Moderate
Arteriolosclerosis	None	None	Moderate	None	Mild	Moderate
Atherosclerosis	None	Mild	Mild	Mild	Moderate	None
Remote infarcts	None	None	None	None	None	None
Remote microinfarcts	None	None	None	None	Yes (*n* = 1)	None
Remote microbleeds	None	None	None	None	None	None
White matter rarefaction	Moderate	Severe	Mild	Moderate	Mild	Moderate
Hippocampal sclerosis	Absent	Absent	Absent	Absent	Absent	Absent

Autoradiography was performed on fresh (unfixed) brain tissue samples of dorsolateral prefrontal cortex and superior temporal cortex which were cut sequentially on a cryostat (Reichert Jung Frigocut 2800 E, Mannheim, Germany) at -20 °C into 10 µm sections and mounted onto silane-coated Histobond + slides (Marienfeld Superior, Lauda-Königshofen, Germany). Blocks were not sectioned exhaustively. Instead, after sectioning, the remainder of each block was processed into tissue homogenate used for ^3^H-MK-6240 (Novandi AB, Södertälje Sweden) binding studies. The slide mounted sections were dried at room temperature for 2 h and stored at -80 °C until used for autoradiography. Sections were immersed in ice-cold 0.01 M sodium phosphate buffer (PBS; pH 7.4) for 30 min, followed by incubation for 1 h at 4 °C in ^3^H-MK-6240 (~ 100,000 cpm/ml). A separate set of sections was evaluated for nonspecific binding by incubating sections in a solution of nonradioactive compound (5 mM MK-6240) mixed with ^3^H-MK-6240. After incubation sections were then rinsed 3 times in ice-cold PBS at 4 °C, air dried, and placed onto a BAS Storage Phosphor screen (GE Healthcare) for 6 days at room temperature. After 6 days of exposure, the Storage Phosphor screens were imaged on a Fuji BAS-5000 phosphorimager at full resolution (25 μm pixels) and the output images were processed and analyzed with ImageJ [41]. Immunohistochemistry for phosphorylated tau pathology (AT8, pSer199/pThr205, Thermo, Waltham, MA; MN1020, lot #R12247424) and the X-34 histofluorescence stain (Dr. WE Klunk, K61-108) were performed as described previously [42, 43] on slide mounted sections adjacent to those used for autoradiography. ^3^H-MK-6240 in-vitro homogenate binding assays to determine K_D_ and B_max_ values of frozen CTE and AD tissues were performed utilizing methods described by Graham et al. [44]

### Part II: In vivo study

#### Participants and study design

The Focused Imaging for the Neurodegenerative Disease Chronic Traumatic Encephalopathy (FIND-CTE) study was a proof-of-concept study evaluating the usefulness of ^18^F-MK-6240 tau PET as a potential biomarker for CTE. The study enrolled 30 male former NFL players who were required to have played 11 + years of organized football (by report using a screening questionnaire), including at least 1 year at the college level and at least 1 season in the NFL as an offensive or defensive lineman, linebacker, tight end, wide receiver, running back, defensive back, or quarterback. Additional inclusion criteria included: age 45–80, native English speaking, ability to provide informed consent or have a legal authorized representative, have an available study partner, able to complete imaging procedures, and no history of moderate to severe TBI or an acute TBI. The former NFL players were also required to have self-reported (not objective testing) cognitive symptoms, based on an AD8 Dementia Screening Interview score of 2 + , at the time of screening [45, 46].

We additionally enrolled four cognitively normal males (59–74 years) without RHI or TBI to complete ^18^F-MK-6240 PET on the same scanners as the former NFL players. These controls were participants in the BU or UCSF ADRCs and were required to have normal cognition, based on intact neuropsychological test performance (i.e., no scores that were > -1.5 SD below normative mean). Besides differences in RHI history and symptomatic status, inclusion criteria were otherwise the same as for the former NFL players. Exclusion criteria for all included contraindications to MRI, MRI scan with evidence of infection or focal lesions or cortical strokes, unstable medical conditions, lifetime history of schizophrenia spectrum disorders, confounding neurological disorders (e.g., brain tumor) or neurodevelopmental conditions, and exposure to investigational agents 30 days prior to study entry, or previous enrollment in a therapeutic trial targeting Aβ or tau.

NFL players and ADRC controls were evaluated at the BU (*n* = 26) or UCSF (*n* = 8) ADRCs, where they underwent a two-day visit that included clinical assessments and interviews, neurological exams, comprehensive neuropsychological testing, self-report neuropsychiatric, RHI, and TBI questionnaires, 3 T MRI, and Aβ (^18^F-Florbetapir) and tau (^18^F-MK-6240) PET. Aβ and tau PET scans were done at least 24 h apart and within one week of each other. A subset of former NFL players (*n* = 4) who had some evidence of tau retention at baseline completed a 13–18 month follow up ^18^F-MK-6240 tau PET scan. All sites received approval by their Institutional Review Board. Participants and/or their legally authorized representative provided written informed consent.

##### Wisconsin Registry for Alzheimer’s Prevention (WRAP)

In order to supplement and enhance the FIND-CTE control group, we included ^18^F-MK-6240 tau PET scans from cognitively unimpaired male controls in WRAP [47], a longitudinal cohort study of middle aged to older adult individuals, of whom 70% reported a parent with a dementia presumed due to AD. We included male participants from WRAP who were Aβ-PET (^11^C-PiB) negative, cognitively normal, and denied a history of traumatic brain injury (*N* = 36, mean [SD] age = 66.3 [6.3]; mean [SD] MMSE = 29.7 [0.5]). History of exposure to RHI in the WRAP cohort is not known. The FIND-CTE and WRAP controls were combined to form one control group (*n* = 40). However, one control from UCSF was excluded from all analyses because they were Aβ-PET positive, resulting in an analytic sample size of 39 controls (eFigure 1 for flowchart).

### Neuropsychological tests and dementia severity

FIND-CTE participants completed a comprehensive neuropsychological battery that assessed attention, visual scanning, and psychomotor speed; verbal and visual learning and episodic memory; executive functions; language; and visuospatial abilities. Tests administered are part of the National Alzheimer’s Coordinating Center (NACC) Uniform Data Set (version 3.0) [48, 49] in addition to supplemental tests. All participants were administered the Test of Memory and Malingering (TOMM) for the detection of sub-optimal effort. We a priori selected measures that assess domains affected by exposure to RHI and CTE, including those that are core features of traumatic encephalopathy syndrome (TES) [50–53], as well as those that are sensitive to the detection of neurodegenerative disease in general. We examined measures of verbal episodic memory (i.e., Neuropsychological Assessment Battery (NAB) List Learning Long Delay Recall, Craft Story Paraphrase and Verbatim Delay Recall, semantic fluency (Animal), and executive functions (F + L Phonemic Fluency, Trail Making Test Part B). The Clinical Dementia Rating (CDR®) Dementia Staging Instrument was used to stage the level of cognitive and functional impairment [54, 55].

### Consensus diagnoses

Multidisciplinary consensus diagnoses were held to adjudicate diagnoses of normal cognition, mild cognitive impairment, and dementia following NACC UDS diagnostic criteria guidelines [48]. During these consensus conferences, we made TES diagnoses (yes or no) and TES level of certainty for underlying CTE pathology, based on the NINDS 2021 consensus diagnostic criteria [53]. TES diagnosis requires: (1) substantial exposure to RHI; (2) impairment in memory and/or executive functioning and/or neurobehavioral dysregulation; (3) a progressive course; and (4) clinical features that are not fully accounted for by other conditions. Suggestive, possible, or probable certainty for CTE pathology is determined based on RHI thresholds, core features, functional status, and supportive features. Demographic, medical, clinical, and neuropsychological data were first presented (blinded to biomarkers) and syndromic diagnoses were made. Following clinical syndrome classification, biomarkers were presented and suspected etiological diagnoses were made.

### Demographic and athletic characteristics

Demographic information was self-reported by all participants, including age (years), sex (male or female), self-reported race (re-coded as non-Hispanic Black versus White) and years of education. The Boston University Repetitive Head Impact Exposure Assessment [56] was administered to all participants to query athletic history and proxies of exposure to RHI, including total seasons of play, age of first exposure to football, and position(s) played.

### Image acquisition

MRIs were conducted using 3 T Siemens Prisma (UCSF), 3 T Philips Ingenia Elition (BU), or 3 T GE Signa 750 (WRAP). All images were acquired at high resolution using 3D sequences, including T1 MPRAGE.

PET/CT scanners included GE Discovery 710 (BU), Siemens Biograph Vision 600 (UCSF), and Siemens ECAT EXACT HR + (WRAP). The ^18^F-Florbetapir protocol included a 370 MBq (10 mCi) bolus injection. A 3D continuous 20-min brain scan acquisition consisting of four five-minute frames was done 50 min post-injection. The use of ^18^F-MK-6240 for BU and UCSF participants in this study was carried out through an Investigational New Drug (IND #153905) from the U.S. FDA, and included a 185 MBq (5 mCi) bolus injection followed by dynamic 3D, 40-min continuous acquisition consisting of 8 × 5 min frames beginning at 70 min post-injection. PET images for the GE Discovery 710 were reconstructed in a 192 × 192 × 47 matrix, compared with 440 × 440 × 159 for the Siemens Biograph Vision 600. Corrections for random coincidences, scatter, system dead time and attenuation were performed as provided by the PET scanner manufacturers. The ^18^F-MK-6240 scan from one NFL player recruited at BU was excluded as the data were not acquired within the 70–90 time window used for analysis, leaving a final analysis cohort of 29 former NFL players (eFigure 1).

^18^F-MK-6240 scans from WRAP were acquired following a 370-MBq bolus injection, and used a 40 min time window starting at 70 min post-injection. The 70-90 min window selection as well as the reconstruction (which included optimized subset expectation maximization, ECAT, version 7.2.2, in a 128 × 128 × 63 matrix) are described previously in detail [15].

### Image processing

T1 MRIs were automatically segmented and parcellated using FreeSurfer version 7.1 (surfer.nmr.mgh.harvard.edu) [57]. Cerebellar subregions were automatically parcellated using the Spatially Unbiased Infratentorial (SUIT) atlas reverse normalized to each participant’s native space MRI, as previously described [58].

Five-minute PET frames were realigned and averaged within the 70-90 min post-injection time window for ^18^F-MK-6240 and 50-70 min time window for Florbetapir. PET images were then coregistered to each participant’s MRI using Statistical Parametric Mapping 12 (SPM12; Wellcome Department of Imaging Neuroscience, Institute of Neurology, London, UK). PET standardized uptake value ratio (SUVr) images were created using inferior cerebellar gray matter for ^18^F-MK-6240 [15] and whole cerebellum for Florbetapir. All SUVr images were smoothed to a final resolution of 8 mm isotropic.

For voxelwise regression analyses, we warped ^18^F-MK-6240 SUVr images to MNI space by estimating the optimal nonlinear transformation between each subject’s T1 MRI and the T1 MRI template in SPM12 Old Normalization, then applying this transformation to the coregistered ^18^F-MK-6240 SUVr image. Warped ^18^F-MK-6240 SUVr images were then masked to include only gray and white matter voxels within the brain, excluding brainstem and cerebellum.

For processing longitudinal ^18^F-MK-6240 scans in 4 NFL players, we used an eroded cerebellar white matter reference region, which has been found to be more sensitive than cerebellar gray matter to detecting subtle changes in signal over time [59].

### PET analysis

#### Visual evaluation

Native-space ^18^F-Florbetapir SUVr images for NFL players and controls scanned were visually read as positive or negative for cortical uptake by a certified and experienced visual rater (GDR), and one UCSF control was excluded from subsequent analysis due to Aβ-PET positivity (Figure S1). WRAP controls were scanned for Aβ pathology with ^11^C-PIB and were all determined to be Aβ negative based on visual read [60, 61]. ^18^F-MK-6240 SUVr images for NFL players were reviewed and qualitatively described by a visual rater (GDR) to identify brain regions with cortical uptake.

### Region-of-interest (ROI) analysis

^18^F-MK-6240 cortical uptake was quantified in native space in 5 bilateral medial temporal lobe (MTL) ROIs and 12 bilateral cortical ROIs based on combinations of Desikan-Killiany atlas ROIs implemented in Freesurfer 7.1 (eTable 1) [57]. We then performed one-tailed t-tests to identify ROIs with higher SUVr in former NFL players than in controls, with age as a covariate: SUVr_ROI_ ~ β_0_ + β_1_ Football Status + β_2_ Age. ROIs with *p* < 0.05 were considered significant, and due to the small sample size and exploratory nature of the analyses, we did not apply correction for multiple comparisons.

### Voxelwise analysis

Voxelwise regressions were then performed in SPM12 on the warped masked ^18^F-MK-6240 SUVr images to identify voxels with higher SUVr in former NFL players than controls, controlling for age: SUVr_voxel_ ~ β_0_ + β_1_ Football Status + β_2_ Age. We evaluated the results using two significance thresholds based on one-tailed t-tests (NFL > Controls): *P* < *0.001* uncorrected for multiple comparisons, and *P* < 0.05 with familywise error (FWE) correction. No cluster size or extent thresholding was used.

### PET associations with RHI and clinical measures

Partial correlations tested associations between ^18^F-MK-6240 ROI SUVr (i.e., frontal, temporal, and MTL) with (1) proxies of RHI (i.e., years of American football play, age of first exposure to football), and (2) neuropsychological raw scores. For analyses with cognitive measures, participants who had suboptimal performance on a standalone measure of effort (TOMM Trial 2) were excluded (*n* = 5). All analyses controlled for age with years of education added as a covariate for models with cognitive measures as outcomes. Total years of football play was included as a covariate for models that examined age of first exposure. Due to the small sample size, emphasis is placed on effect sizes and *p* < 0.10 are reported as potentially meaningful. *P*-values are not adjusted for multiple comparisons given the small sample size and limited statistical power.

## Results

### Part I: Autoradiography and in-vitro binding

The six autopsy cases had CTE stage III (of IV) and CERAD neuritic Aβ plaque score of 0. Four of these cases also had Braak NFT Stage III (A0, B2, C0), and two cases had Braak NFT Stage III and Thal Phase 1 (A1, B2, C0) (Table 1). Three cases had TDP-43 pathology in medial temporal lobe structures and one had cerebral amyloid angiopathy. There were otherwise no co-morbid neurodegenerative disease diagnoses. Immunohistochemical analysis of frozen sections adjacent to those used for autoradiography studies and from the same frozen tissue block used for the binding assay showed that case 6 had moderate to frequent accumulation of p-tau in the frozen frontal and temporal cortical samples. Cases 1–4 had sparse p-tau accumulation (none in the frontal cortex sample for case 3) and case 5 had moderate p-tau aggregation in the temporal cortex sample but only sparse in the frontal cortex sample.

**Fig. 1:**
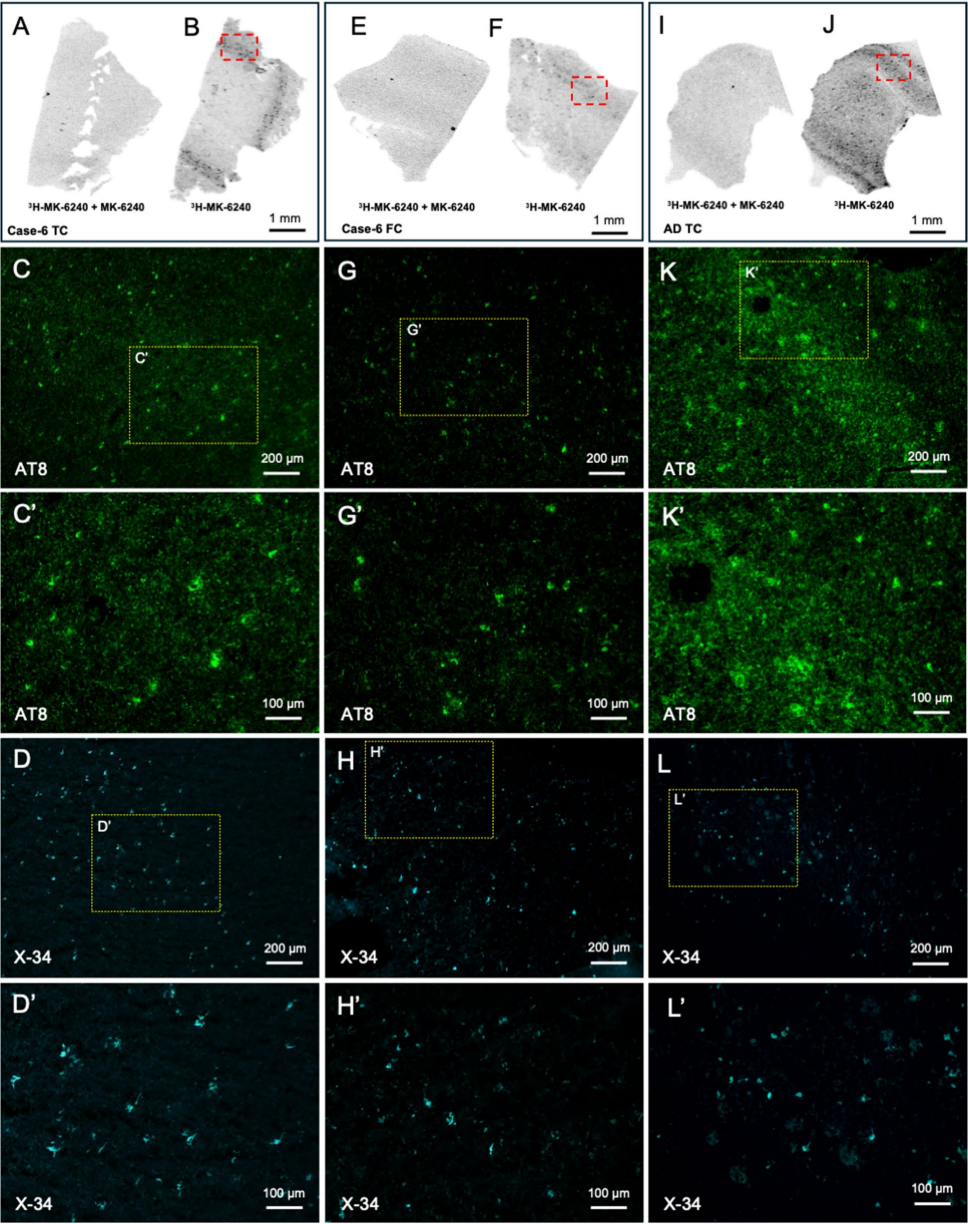
^3^H-MK-6240 autoradiography, AT8 immunohistochemistry, and X-34 histology in a CTE Stage III case and an AD Braak NFT Stage VI case. ^3^H-MK-6240 autoradiography with (**A**, **E**, **I**) and without (**B**, **F**, **J**) cold compound block, AT8 immunohistochemistry (green; C–C’, G-G’, K-K’) and X-34 stain (blue; D-D’, H–H’, L-L’) on three adjacent 10 μm cryosections of superior temporal cortex (left column) and dorsolateral prefrontal cortex (middle column) from a case neuropathologically diagnosed as CTE Stage III, with Braak NFT Stage III and Thal-1 phase for amyloid-β (Case 6) and superior temporal cortex from an AD case with Braak NFT Stage VI (right column). Low power magnification images of AT8 (**C**, **G**, **K**) and X-34 (**D**, **H**, **L**) illustrate areas with high densities of labeled structures (marked by the red boxes in **B**, **F**, **J**). Panels C’, **G**’, **K**’ and **D**’, **H**’, **L**’ are higher magnifications of the areas delineated by the yellow boxes in **C**, **G**, **K** and **D**, **H**, **L**, respectively. FC = dorsolateral prefrontal cortex; TC = superior temporal cortex. Scale bars: 1 mm (**A**, **B**, **E**, **F**, **I**, **J**); 200 μm (**C**, **D**, **G**, **H**, **K**, **L**), 100 μm (**C**’, **D**’, **G**’, **H**’, **K**’, **L**’)

Figure 1 shows ^3^H-MK-6240 autoradiography, pSer202/pThr205 tau immunofluorescence (AT8), and X-34 stain of fibrillar aggregates in sections of temporal and frontal cortex from a CTE case with Thal Phase 1 and Braak NFT Stage III (case six, Table 1), and positive control sections of temporal cortex from a typical AD case with Braak NFT Stage VI. Of the CTE cases examined in this study, only case 6 (Low AD Neuropathologic Change: A1, B2, C0, Table 1) had a laminar pattern of ^3^H-MK-6240 autoradiography signal that was distinct in the superior temporal cortex and less so in the dorsolateral frontal cortex, spatially corresponding to AT8-immunoreactive and X-34 labeled tau lesions on adjacent sections, and was not present in the self-blocked sections. However, the ^3^H-MK-6240 autoradiography signal and p-tau immunostaining in the superior temporal cortex of CTE case six were substantially lower than in the superior temporal cortex from the AD case. The other CTE cases had ill-defined ^3^H-MK-6240 autoradiography signal and sparse to moderate tau pathology in tissue sections immediately adjacent to those used for autoradiography.

For the in-vitro binding analysis, case 6 (CTE/Thal Phase 1, illustrated in Fig. 1) had a K_D_ = 2.0 ± 0.9 nM, B_max_ = 97 ± 24 nM (*n* = 3) compared with the AD case that had a K_D_ = 0.46 ± 0.12, B_max_ = 378 ± 84 nM (*n* = 4).

### Part II: In vivo study

Table 2 provides an overview of participant characteristics. On average, the former NFL players were 58.9 years old (SD = 7.8) and 13 (43.3%) identified as Black. As part of study eligibility criteria, all former NFL players had self-reported cognitive symptoms at time of study screening. However, results of the diagnostic consensus conferences that integrated *objective* neuropsychological test data and used established diagnostic criteria revealed that nine (30.0%) had normal cognition, 16 (53.3%) had MCI or cognitive impairment-not MCI, and 5 (16.7%) had dementia. Twenty-two (73.3%) met criteria for TES, including 6 (20.0%) of whom had TES-CTE possible and 11 (36.7%) had TES-CTE probable.

**Table 2:** Sample characteristics of 30 symptomatic former national football league players

Demographics	Former NFL Players *N* = 30	Controls *N* = 40
Age, mean (SD) years	58.9 (7.8)	65.7 (6.3)
Education, mean (SD) years	16.6 (1.8)	17.3 (3.0)
Sex, n (%) male	100	100
Race		
Black, n (%)	13 (43.3)	1 (2.5)
White, n (%)	17 (56.7)	39 (97.5)
**Athletics**		
Total years of football, mean (SD)	18.3 (4.3)	–
Total years played in the NFL, mean (SD)	7.3 (3.9)	–
Age of first exposure to football, mean (SD)	11.3 (2.4)	–
Primary position in the NFL		–
Tackle, n (%)	3 (10.0)	
Center, n (%)	3 (10.0)	
Tight End, n (%)	3 (10.0)	
Full Back, n (%)	2 (6.7)	
Running Back, n (%)	1 (3.3)	
Wide Receiver, n (%)	1 (3.3)	
Defensive Tackle, n (%)	2 (6.7)	
Other Defensive Lineman, n (%)	1 (3.3)	
Middle Linebacker, n (%)	2 (6.7)	
Other Linebacker, n (%)	4 (13.3)	
Cornerback, n (%)	4 (13.3)	
Safety, n (%)	2 (6.7)	
Free Safety, n (%)	1 (3.3)	
Other Defensive Back, n (%)	1 (3.3)	
**Diagnosis**		
Traumatic Encephalopathy Syndrome, n (%)	22 (73.3)	–
Level of CTE certainty		–
Suggestive, n (%)	5 (16.7)	–
Possible, n (%)	6 (20.0)	–
Probable, n (%)	11 (36.7)	–
Cognitive Diagnosis		
Cognitively normal, n (%)	9 (30.0)	100
MCI Amnestic, single domain, n (%)	4 (13.3)	0
MCI Amnestic, multiple domains, n (%)	5 (16.7)	0
MCI Non-amnestic, single domain, n (%)	1 (3.3)	0
MCI Non-amnestic, multiple domains, n (%)	3 (10.0)	0
Cognitively impaired, not MCI, n (%)	3 (10.0)	0
Dementia, n (%)	5 (16.7)	0
Global CDR score		–
0	7 (23.3)	–
0.5	18 (60.0)	–
1.0	5 (16.7)	–
CDR sum of boxes, mean (SD)	1.78 (1.77)	–
**Elevated 18F-Florbetapir, n (%)**	0	1 (2.5)

Note. One NFL player was excluded from analyses due to PET acquisition reasons. One control was excluded from analyses because they were amyloid PET positive. RHI history in WRAP controls is unknown. Controls include those from WRAP (*n* = 36) and BU and UCSF (*n* = 4)

*Abbreviations*: *CTE* chronic traumatic encephalopathy, *MCI* mild cognitive impairment, *PET* positron emission tomography

### ^18^F-Florbetapir PET

All 30 of the former NFL players had a negative Florbetapir PET on visual read. Only 1 of the 4 same scanner controls had a positive Florbetapir PET with a centiloid value of 59. As previously mentioned, this control was excluded from analyses (Figure S1). Aβ status of the WRAP cognitively normal controls was negative by visual read.

### ^18^F-MK-6240 tau PET

**Fig. 2:**
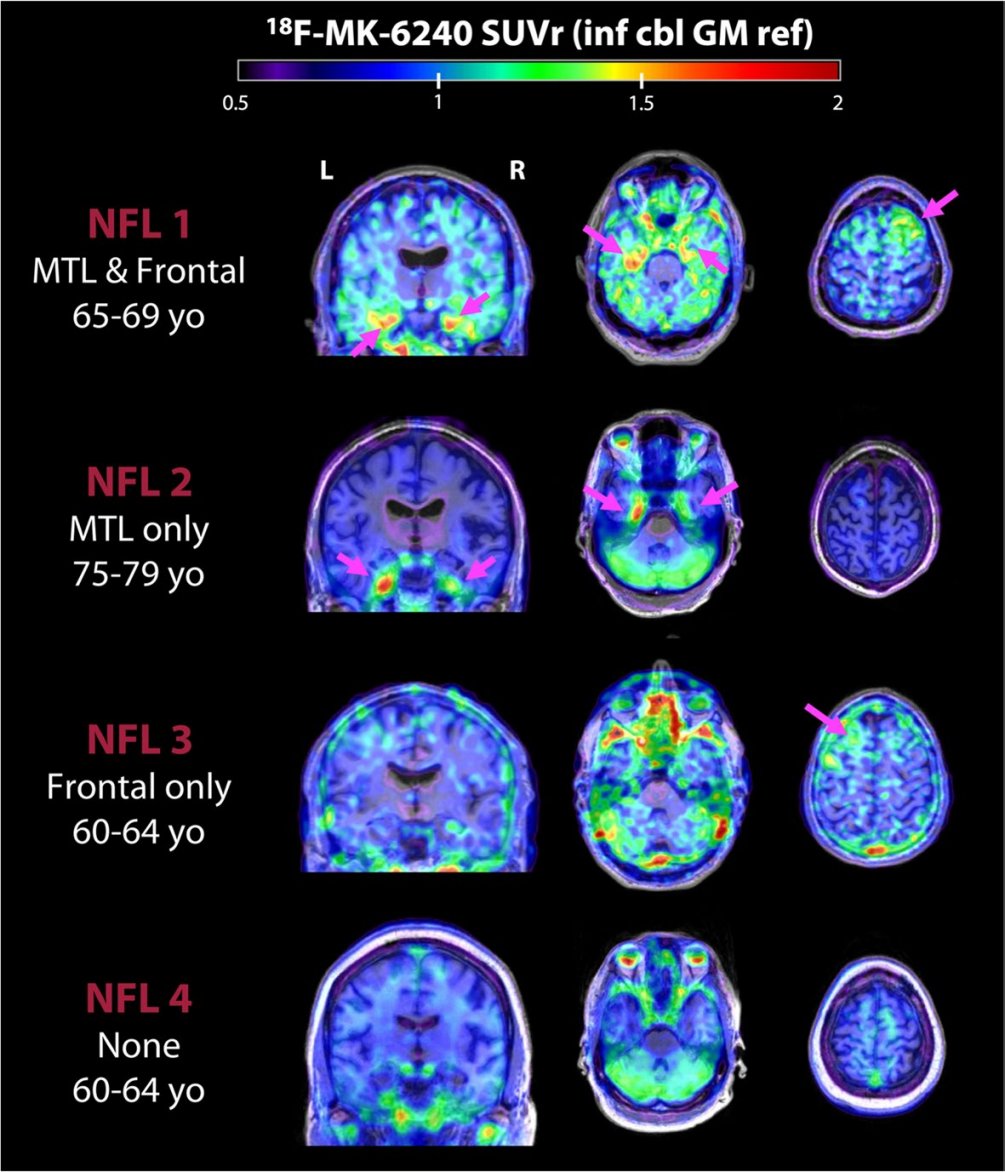
Example ^18^F-MK-6240 scans in four former NFL players. Each row shows data from a single participant. Image slices depict 70-90 min SUVr, referenced against inferior cerebellar gray matter and overlaid on native space MRIs. Pink arrows point to areas with elevated cortical signal according to visual read

**Fig. 3:**
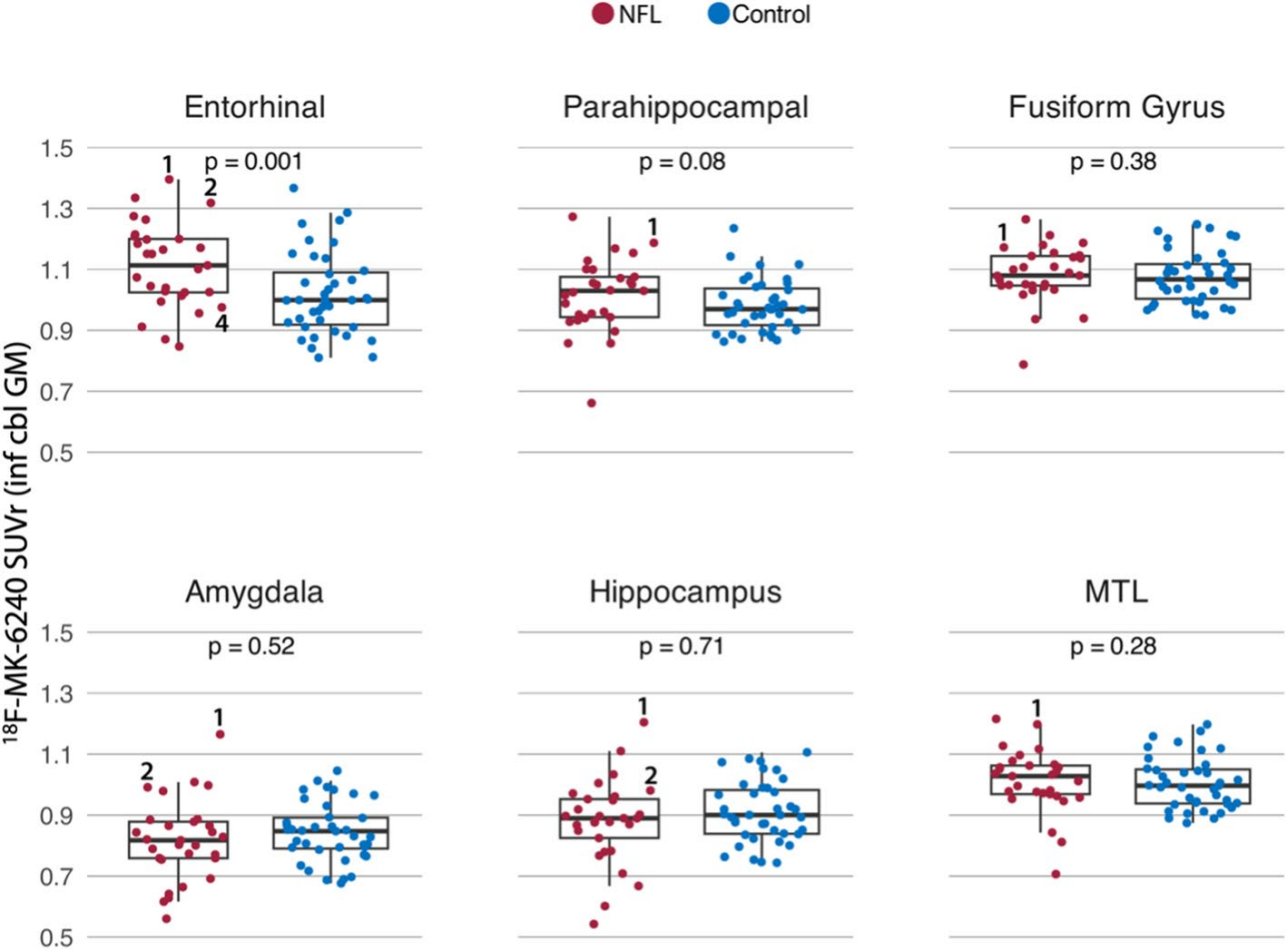
^18^F-MK-6240 SUVr in medial temporal lobe regions. Each subplot shows the distribution of mean SUVr values for 29 NFL players (red points) and 39 cognitively unimpaired controls (blue points) in the bilateral entorhinal cortex, parahippocampal gyrus, fusiform gyrus, amygdala, hippocampus, and MTL (all five regions combined, weighted by volume), respectively. *P*-values correspond to values from a one-tailed *t-*test (patients > controls) in a multiple linear regression predicting SUVr as a function of diagnostic group and age. Scatter points from individual participant scans in Fig. 2 are labeled if they were outside the first and third quartiles (i.e. outside the box) for each region. For example, label “1” corresponds to Fig. 2 NFL 1

**Fig. 4:**
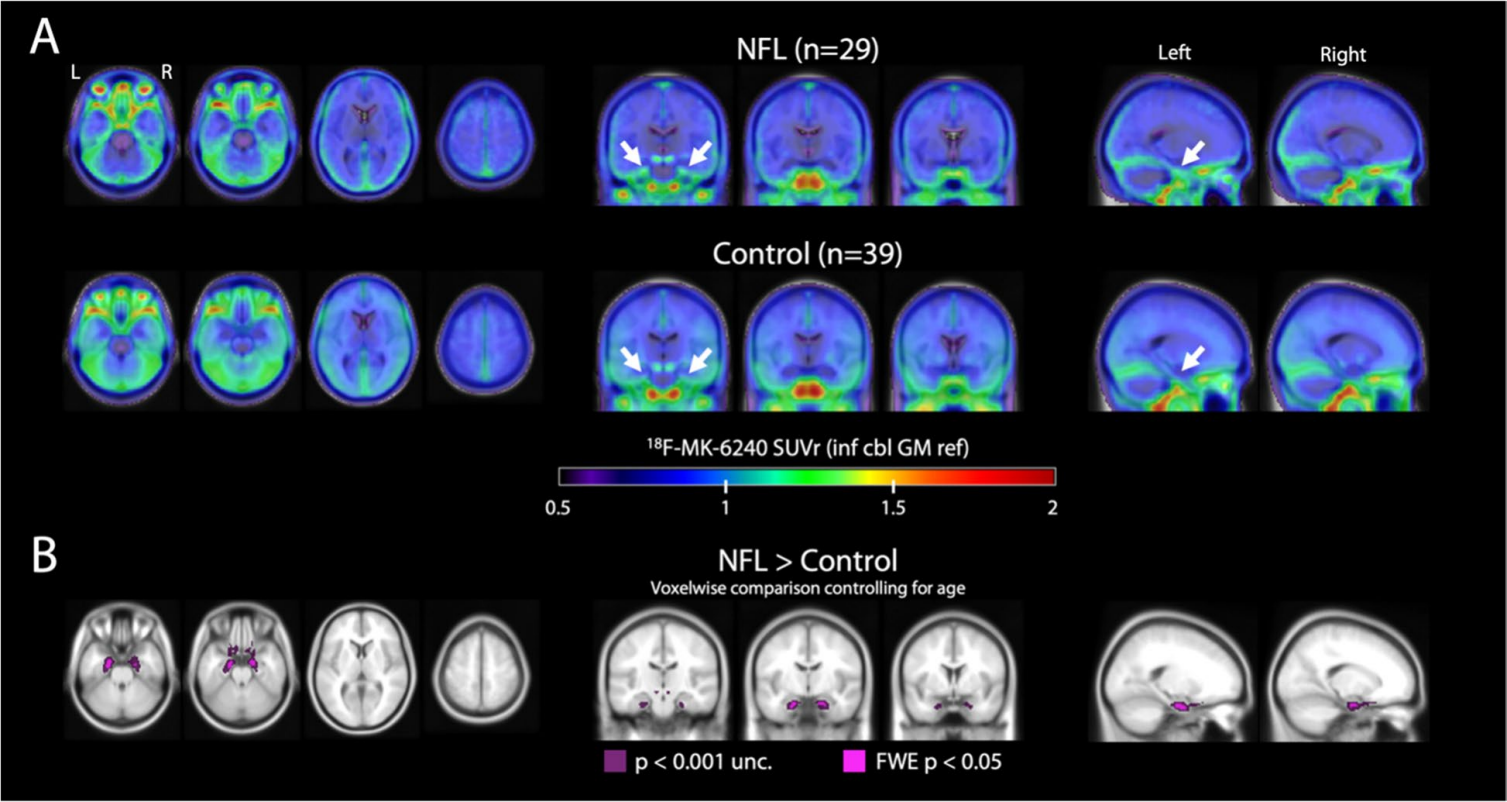
Voxelwise statistics. **A** Voxelwise mean across ^18^F-MK-6240 SUVr from 29 NFL participants (top) and 39 cognitively unimpaired controls (bottom), transformed to MNI space and scaled identically. White arrows point to MTL regions with visually greater signal in NFL participants versus controls. **B** Voxelwise regression results show voxels within the brain for which SUVr in ex-NFL players exceeded controls at two statistical thresholds (violet = *p* < 0.001, uncorrected for multiple comparisons; pink = *p* < 0.05, family-wise error corrected) with no cluster thresholding applied

Overall, there was variable ^18^F-MK-6240 uptake across participants, including 16 with no cortical signal, 7 with medial temporal lobe uptake, 2 showing focal frontal uptake, and 4 showing both medial temporal and frontal uptake. Characteristics of individuals with uptake are shown in eTable 2. Figure 2 provides illustrative case examples of the main patterns found in this study. Patient 1 is a former NFL player between 65–69 years old who has MCI (amnestic, single domain) and is TES-CTE possible. This patient had both MTL and superior frontal uptake, consistent with the expected distribution of p-tau neuropathology of CTE. Patient 2 is a 75–79 years-old former NFL player who has MCI (amnestic, single domain) and is TES-CTE suggestive. He had only MTL uptake. Patient 3 is a 60–64 year old who is cognitively normal and is TES-CTE suggestive; he had only frontal uptake. Patient 4 had no uptake but is a 60–64 year old who has dementia and is TES-CTE probable; note that he also had a Lewy body syndrome. Patterns were variable in each participant in terms of spread and intensity. Off-target meningeal binding, present in various intensities across all scans, made it challenging to distinguish signal on the cortical surface from meningeal binding in some instances (eFigure 2). Across all participants in the study, signal intensity was low-intermediate in most regions of interest (range SUVr = 0.49 – 1.40, Figs. 3 and 4), highest in the entorhinal cortex.

At the group level, linear regression models examining native space ^18^F-MK-6240 SUVr in selected ROIs as a function of group (former NFL player or control) and age showed that former NFL players had significantly higher SUVr than controls in the entorhinal cortex (NFL: 1.11 ± 0.14; Control: 1.02 ± 0.14 [mean ± SD]; d = 0.86; *p* = 0.001, one-sided t-test) and parahippocampal gyrus (NFL: 1.02 ± 0.12; Control: 0.98 ± 0.09 [mean ± SD]; d = 0.39; *p* = 0.08, trend) (Fig. 3). There were no statistically significant group differences for the other subcortical and cortical regions (all *p* > 0.05; eFigure 3).

Mean SUVr images in former NFL players and controls are shown in Fig. 4A. Voxelwise regressions between these groups concurred with ROI analyses, showing significantly higher uptake in NFL players compared to controls only in two bilateral clusters in the anterior MTL, centered around the entorhinal cortex (Fig. 4B). Both of these clusters survived stringent statistical thresholding at *p* < 0.05 correcting for FWE.

### Longitudinal ^18^F-MK-6240 Scans

**Fig. 5:**
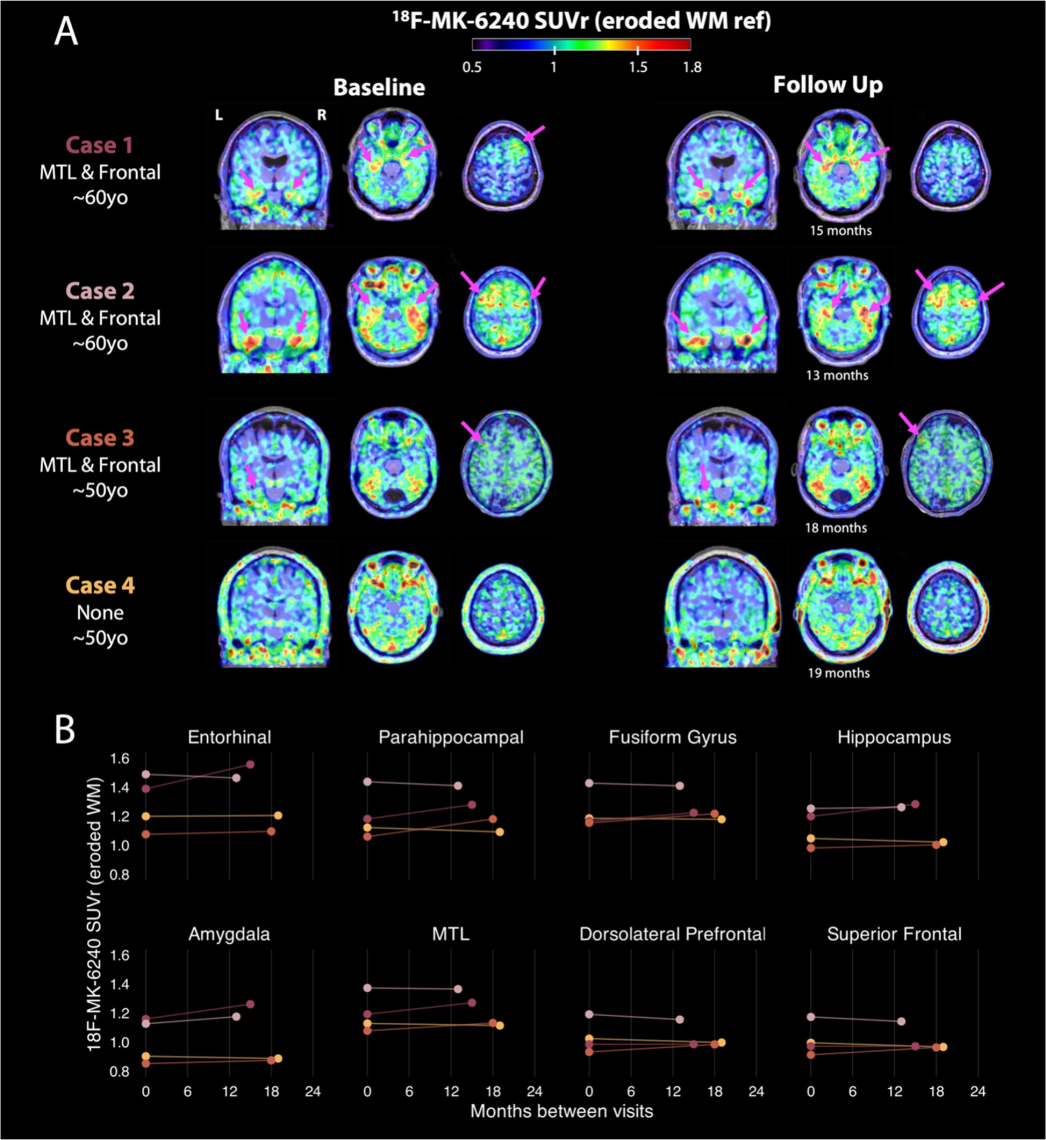
^18^F-MK6240 longitudinal scans in four former NFL players. **A** Each row shows longitudinal data from a single participant. Scans shown depict 70-90SUVr, referenced against eroded white matter and overlaid on native space MRIs. Image slices on the left show the baseline visit, while scans on the right depict a follow-up visit. Time to follow up for each scan is described for each subject below its corresponding axial cut. “Case 1” subject corresponds to the “NFL 1” shown in Fig. 2. **B** Each subplot shows the distribution of mean SUVr values in regions-of-interest visually read as having on-target uptake for 4 former NFL players with longitudinal tau scans. Scatter points represent each individual participant scans, and the line connects a subject on its baseline and follow up visit

Four of the former NFL players had a 13–18-month follow-up ^18^F-MK-6240 scan (Fig. 5). The overall ^18^F-MK-6240 uptake patterns remained stable over time for all four participants.

### Proxies of RHI and.^18^F-MK-6240

Years of football play (*r* = -0.27 to *r* = -0.09) and age of first exposure to football (*r* = -0.29 to *r* = 0.05) were not significantly correlated with SUVr of any ROIs (Table 3).

**Table 3:** Partial correlation matrix of associations between SUVr ROIs and neuropsychological measures and proxies of repetitive head impacts

**ROI**	Total Years of Football Play	Age of First Exposure to Football	NAB List Learning Long Delay	Craft Story Delayed Verbatim	Craft Story Delayed Paraphrase	Trails B	Animal Fluency	Total F & L Words
Entorhinal	-0.17	0.05	-0.16	-0.13	-0.19	0.00	-0.13	0.08
Parahippocampal	-0.08	-0.09	-0.18	-0.38*	-0.40*	0.29	-0.28	0.05
Amygdala	0.09	-0.23	-0.10	-0.10	-0.14	0.03	-0.12	-0.03
Hippocampus	0.04	-0.24	-0.08	-0.19	-0.26	0.18	-0.10	0.02
Inferior Frontal	-0.20	-0.18	0.19	-0.02	-0.01	0.06	-0.47**	0.20
Dorsolateral Prefrontal	0.00	-0.29	0.15	0.08	0.08	-0.05	-0.38*	0.27
Ventromedial Prefrontal	-0.11	-0.21	0.12	0.01	-0.02	-0.05	-0.48**	0.17
Superior Frontal	0.01	-0.28	0.14	0.12	0.12	-0.14	-0.32	0.30
Lateral Temporal	-0.27	-0.19	0.25	0.02	-0.01	0.04	-0.43**	0.12
Superior Temporal	-0.10	-0.21	0.10	-0.15	-0.17	0.20	-0.42**	0.03

All analyses controlled for age. Analyses for clinical measures also controlled for education. Analyses for age of first exposure to football also controlled for total years of football play. *P*-values are not adjusted for multiple comparison given the reduced statistical power

^**^ *p* < 0.05

^*^ *p* < 0.10

### Neuropsychological correlates of.^18^F-MK-6240

After exclusion of former NFL players who had sub-optimal effort on the TOMM, the analytic sample size for SUVR-clinical associations was 24. Neuropsychological test raw scores were normally distributed. There were no significant associations between years of education and the neuropsychological raw scores (ps > 0.10 for all) likely due to range restriction (all but 3 participants had 16 years or more of education). Higher parahippocampal gyrus SUVr was associated with lower Craft Story 21 Delayed Recall paraphrase (*r* = -0.40, *p* = 0.06) and verbatim scores (*r* = -0.38, *p* = 0.08) (Table 3). Lower Animal fluency scores correlated with higher SUVr in the inferior frontal gyrus (*r* = -0.47, *p* = 0.03), ventromedial prefrontal (*r* = -0.48, *p* = 0.02), dorsolateral prefrontal (*r* = -0.38, *p* = 0.08), lateral temporal (*r* = -0.43, *p* = 0.05), and superior temporal (*r* = -0.42, *p* = 0.05) regions. There were no other significant associations between regional SUVr and cognitive test performance.

## Discussion

We evaluated ^18^F-MK-6240 tau PET as a potential biomarker to detect underlying CTE p-tau pathology in symptomatic older former NFL players who are at increased risk for CTE and were Aβ-PET negative. We also conducted an in-vitro ^3^H-MK-6240 binding assay, ^3^H-MK-6240 autoradiography, p-tau immunohistochemistry, and X-34 stain in the same frozen blocks of cortical tissue from six autopsy cases with neuropathological diagnosis of CTE Stage III (of IV) and Braak NFT Stage III (of VI) but no Aβ pathology by CERAD criteria and from a typical AD case with Braak Stage VI. Among the six cases with autopsy-confirmed stage III CTE, clear signal on ^3^H-MK-6240 autoradiography and high binding affinity (K_D_ = 2.0) were observed only in one case with Thal Phase 1, Braak NFT Stage III, and CERAD score of 0 (AD Neuropathologic Change: A1, B2, C0). Binding affinity in this CTE case was less than the comparison AD case (K_D_ = 0.46). ^3^H-MK-6240 autoradiography signal had a laminar distribution which corresponded well to the distribution pattern of tau-immunoreactive lesions in this case, but it appeared less prominent than observed in the comparison AD case (Fig. 1). The other CTE cases had sparse p-tau pathology and lacked a clear ^3^H-MK-6240 ARG signal. The in vivo study of subjectively symptomatic Aβ-PET negative former NFL players showed variable, low-medium intensity ^18^F-MK-6240 signal across participants, including many with no cortical signal (*n* = 16), some with MTL uptake (*n* = 7), and some showing focal frontal uptake (*n* = 2). A subset had both MTL and frontal ^18^F-MK-6240 uptake (*n* = 4). SUVr was highest in the entorhinal cortex (NFL: 1.11 ± 0.14 [mean ± SD]). At the group level, former NFL players had greater ^18^F-MK-6240 uptake in the entorhinal cortex and the entire parahippocampal gyrus compared with the control group. There were no group differences for the cortical ROIs and there were various levels of off-target meningeal binding in all cases. Among the four former NFL players who had a 13–18 month follow up scan, ^18^F-MK-6240 signal was reproducible and stable.

Postmortem autoradiography and in-vitro binding studies of tau PET tracers are essential to guide the conduct and interpretation of ^18^F-MK-6240 in human studies, including their specificity to p-tau. Previous autoradiography studies of ^3^H-MK-6240 in brain tissue from CTE Stage II/III to IV and in other non-AD neurodegenerative diseases have been negative [24, 29]. While one ^3^H-MK-6240 study found binding in tissue with severe CTE (stage IV), it was in the presence of AD co-pathology [30]. Collectively, these reports are in agreement with our results. We observed clear autoradiography signal and high binding affinity only in one of the six cases with autopsy-confirmed stage III/IV CTE and no neuritic plaque pathology by CERAD criteria, however, this case also had Thal Phase 1 and Braak NFT Stage III. In an adjacent tissue section analyzed by AT8 immunohistochemistry, there were no perivascular or deep sulcal p-tau patterns typical of CTE, and the clearly laminar pattern of p-tau immunoreactive lesions in the superior temporal cortex of this case could be due to a coexisting AD tauopathy. In support of this, the binding affinity and binding density in this and other CTE cases were less relative to a typical AD case. This is not unexpected given ^18^F-MK-6240 is optimized for binding to AD-related NFTs and binding affinity is diminished in non-AD neurodegenerative diseases [24, 29]. This could be related to differences in the molecular composition and/or density of p-tau in CTE and other non-AD tauopathies [7–9, 62]. Although both CTE- and AD-related p-tau lesions are a mix of three (3R) and four (4R) microtubule binding site repeat motifs, CTE tau has been reported as 4R predominant in mild cases and shift to 3R predominant as the disease progresses to more severe stages [7]. Furthermore, the CTE tau fibril folding pattern is conformationally different from AD tau and has a unique morphology [9, 63]. The current study indicates that the density of p-tau in CTE Stage III is lower compared to AD and this could introduce challenges for in vivo detection using currently available tau PET tracers. Indeed, in most autopsy cases in our study the density of AT8-immunoreactive p-tau assessed in tissue sections adjacent to those used for autoradiography was low and perhaps not sufficiently developed to produce an autoradiography signal. This idea is supported by FTP tau PET imaging studies reporting that positivity is most reliably observed in cases at Braak Stages V and VI [19, 64], though MK-6240 may be able to detect earlier Braak Stages [65]. Note that while all cases were determined to be CTE stage III, the CTE staging scheme is in part dependent on the location of p-tau rather than the overall burden of pathology.

The autoradiography and in-vitro binding results complement our study of ^18^F-MK-6240 tau PET in human participants. In some individuals and at the group level, there was elevated ^18^F-MK-6240 uptake in the entorhinal cortex and parahippocampal gyrus among the former NFL players. This observation is less likely to be related to AD given the entire analytic sample was Aβ-PET negative. The MTL PET signal could reflect in vivo tracer binding to underlying CTE tau pathology. CTE is characterized by p-tau in neurons around small blood vessels with initial deposition (low stage I/II) in the cortex (frontal, temporal) and MTL involvement in later disease stages (high stage III/IV) [4–6]. However, recent data shows a cortical sparring subtype of CTE characterized by predominant p-tau accumulation in the MTL with less involvement of the cortex (note that the perivascular p-tau lesion is still present and required) [66]. P-tau density has been described to be highest in the entorhinal cortex including in stage I/II [6]. The intensity of MTL ^18^F-MK-6240 signal observed in the former NFL players was low in comparison to what is observed in AD [15, 67]. Binding to primary age-related tauopathy (PART), or binding to tau-negative neurodegeneration in limbic-predominant age-related TDP-43 (LATE) are possible alternative explanations for the observed ^18^F-MK-6240 signal, but may be less likely due to the relatively young age of the sample and the reported high specificity of ^18^F-MK-6240 for tau pathology [29]. Similar to in-vitro binding studies, ^18^F-MK-6240 tau PET signal intensity is diminished in non-AD neurodegenerative diseases [15, 68].

At the individual level, a subset of former NFL players had low intensity ^18^F-MK-6240 uptake in the frontal cortex ± MTL. This distribution mimics the neuropathological descriptions of stage III/IV CTE [4–6]. The signal was variable, intensity was low, and there was no group effect for cortical ROIs. [59, 69]. In low stage CTE (stage I/II), cortical CTE p-tau deposits are focal, patchy, and located at the depths of the cortical sulci. A similar pattern has been described for other tracers. Flortaucipir has been shown to differentiate symptomatic former NFL players from controls in cortical ROIs at the group level but effect sizes are small and this finding is not consistent across studies [17, 20, 21]. Detecting low stage CTE may be challenging with PET given the focal and low overall density of pathology, as well as the location at sulcal depths, regions where PET signal is diluted by partial volume effects from adjacent CSF. A specific limitation of the ^18^F-MK-6240 radiotracer is frequent and sometimes high intensity meningeal uptake, which can be difficult to disentangle visually or quantitatively from intraparenchymal signal at the interface between cortex and meninges. ^18^F-MK-6240 (and other tau PET tracers) may have increased ability to detect MTL and high stage CTE (stage III/IV) because p-tau NFTs are more well-formed, contain a mix of 3R/4R, and the pathology is not as patchy and focal as cortical pathology.

The primary risk factor for CTE neuropathology is exposure to RHI, for example from American football [1, 70]. We found minimal associations between metrics of exposure to RHI (years of football play, age of first exposure) and ^18^F-MK-6240 uptake. This could be due to lack of statistical power from the small sample size. Range restriction is an alternative explanation. Our sample included all highly exposed individuals who played at the same level of play (professional). When lower-level football players (e.g., youth, high school, college) are included, there is a dose–response relationship for both years and level of play with risk for CTE neuropathology [2, 71]. Players with more than 14.5 years of play are 10 times more likely to have CTE compared with those who played fewer than 14.5 years (all but 2 participants in this sample had more than 14 years of play) [2]. Regarding clinical associations, the former NFL players spanned the clinical continuum (normal cognition, MCI, dementia) and parahippocampal and frontotemporal ^18^F-MK-6240 uptake were associated with worse episodic and semantic memory, respectively. Among former NFL players, memory is a frequently impaired domain and a core feature of the TES research diagnostic criteria [51–53]. It is reasonable to conclude that the observed ^18^F-MK-6240 MTL signal might be capturing mild but clinically-meaningful pathology contributing to memory loss. Whether this is due to CTE or superimposed AD tau pathology is unknown.

Tau radiotracers developed for AD are proving to have restricted applicability for the detection of CTE p-tau aggregates. There needs to be discovery efforts for the development of tau radiotracers that have specificity to CTE p-tau. Furthermore, the biochemical composition of tau inclusions evolve from containing primarily 4R tau early in the disease course, to primarily 3R tau as the disease advances [62]. Therefore, a given PET tracer may show differential affinity for tau inclusions in early vs. later-stage CTE, exacerbating the challenge of i*n vivo*detection. Imaging of the CTE p-tau pathology will also be challenging for reasons previously enumerated, including the density and location of pathology. Blood based biomarkers should be pursued in parallel as plasma assays of different p-tau epitopes now exist and can aid in differential diagnosis. We observed small effects for p-tau181 and ptau231 in discriminating former professional football players and asymptomatic non-RHI men [72]. Identification of the specific epitopes affected in CTE tissue will be needed to guide plasma p-tau epitope assay targets.

The current PET study has several limitations. The sample size was small and consisted of all males. Our findings lack generalizability to other populations including football players who played at amateur levels. The control group was predominantly from the WRAP and scanner differences across sites might have influenced the results. The groups were also not well-matched in terms of age, race, and other characteristics. It is also unknown if the WRAP controls had exposure to RHI from contact and collision sports or other sources as this was not assessed; however, none of them reported a history of TBI. Evaluation of potential biomarkers for CTE is complicated by recruitment challenges. CTE can only be diagnosed post-mortem and former NFL players were recruited because autopsy studies have shown this population to be at greatest risk for CTE [2, 73]. However, not all NFL players will develop CTE and 30% of our sample had normal cognition and ~ 50% of the sample was less than 60 years old. It is possible that many of the former NFL players did not have meaningful enough pathology to be detected by tau PET. Future research that correlates MK-6240 with other established biological measures of disease severity (e.g., atrophy on MRI) will help to place the current findings in context.

## Conclusions

We present evidence of ^3^H-MK-6240 binding to post-mortem CTE tissue and in vivo PET uptake in a subset of high-risk participants. Among the subset of participants who had in vivo evidence of ^18^F-MK-6240 uptake, tau PET retention was most frequently observed in MTL structures, and this region is often most affected in high stage CTE. Thus, ^18^F-MK-6240 PET may have usefulness for detecting late stages of CTE, and possibly as a screen for coexisting AD p-tau pathology. Larger studies with longitudinal follow-up to autopsy in cases representing all CTE stages, with and without AD co-pathology, are needed to further evaluate the utility of ^18^F-MK-6240 PET as a biomarker of CTE.

## Supplementary Information

Supplementary Material 1.

## Data Availability

Data generated and/or analyzed during the current study are available from the corresponding author upon reasonable request. Aspects of the data are also available in the publicly available National Alzheimer’s Coordinating Center data set.

## Abbreviations

Aβ: Amyloid-beta
AD: Alzheimer’s disease
ARTAG: Aging-related tau astrogliopathy
BU: Boston University
CTE: Chronic traumatic encephalopathy
FTP: Flortaucipir
MRI: Magnetic resonance imaging
MTL: Medial temporal lobe
NFL: National Football League
PET: Positron emission tomography
p-tau: Hyperphosphorylated tau
RHI: Repetitive head impacts
SUVr: Standardized uptake value ratio
UCSF: University of California San Francisco
WRAP: Wisconsin Registry for Alzheimer’s Prevention
