# Supplementary material for: 18F-MK-6240 tau PET in patients at-risk for chronic traumatic encephalopathy
Source: Mol Neurodegener. 2025 Feb 25;20:23. doi: 10.1186/s13024-025-00808-1 (PMC11852567; doi:10.1186/s13024-025-00808-1)

**Supplementary Online Content**

**eTable 1. Freesurfer labels used to define ROIs expected to accumulate CTE pathology**

**eFigure 1. Study flowchart**

**eFigure 2. ^18^F-MK-6240 SUVr case with visual off-target binding conflicting with cortical binding**

**eFigure 3. ^18^F-MK-6240 SUVr in regions outside the MTL**

**eTable 1. Freesurfer labels used to define ROIs expected to accumulate CTE pathology**

**eTable 2.** Sample Characteristics of 13 Symptomatic Former National Football League Players who had 18F-MK-7240 Uptake

| **Demographics** | **MTL Only Uptake**  **N= 7** | **Frontal Only Uptake**  **N=2** | **MTL + Frontal Uptake**  **N = 4** |
| --- | --- | --- | --- |
| Age, mean (SD) years | 61.3 (10.1) | 56.0 (5.7) | 60.8 (3.8) |
| Education, mean (SD) years | 16.4 (1.6) | 17.0 (1.4) | 18.3 (3.3) |
| Race, n (%) Black | 3 (42.9) | 0 | 2 (50.0) |
| **Athletics** | |  | |
| Total years of football, mean (SD) | 16.3 (3.8) | 18.5 (0.7) | 17.8 (1.7) |
| Total years played in the NFL, mean (SD) | 6.6 (2.9) | 3.0 (2.8) | 6.5 (1.3) |
| **Diagnosis** | |  |  |
| Traumatic Encephalopathy Syndrome, n (%) | 6 (85.7) | 2 (100.0) | 4 (100.0) |
| Level of CTE certainty | | | |
| Suggestive, n (%) | 3 (42.9) | 1 (50.0) | 0 |
| Possible, n (%) | 0 | 0 | 3 (75.0) |
| Probable, n (%) | 3 (42.9) | 1 (50.0) | 1 (25.0) |
| Cognitive Diagnosis | |  | |
| Cognitively normal, n (%) | 3 (42.9) | 1 (50.0) | 0 |
| MCI Amnestic, single domain, n (%) | 2 (28.6) | 0 | 1 (25.0) |
| MCI Amnestic, multiple domains, n (%) | 1 (14.3) | 1 (50.0) | 1 (25.0) |
| MCI Non-amnestic, single domain, n (%) | 0 | 0 | 0 |
| MCI Non-amnestic, multiple domains, n (%) | 0 | 0 | 1 (25.0) |
| Cognitively impaired, not MCI, n (%) | 0 | 0 | 0 |
| Dementia, n (%) | 1 (14.3) | 0 | 1 (25.0) |

**Note**. Abbreviations: MTL = medial temporal lobe; MCI = mild cognitive impairment

**eFigure 1.** Study flowchart
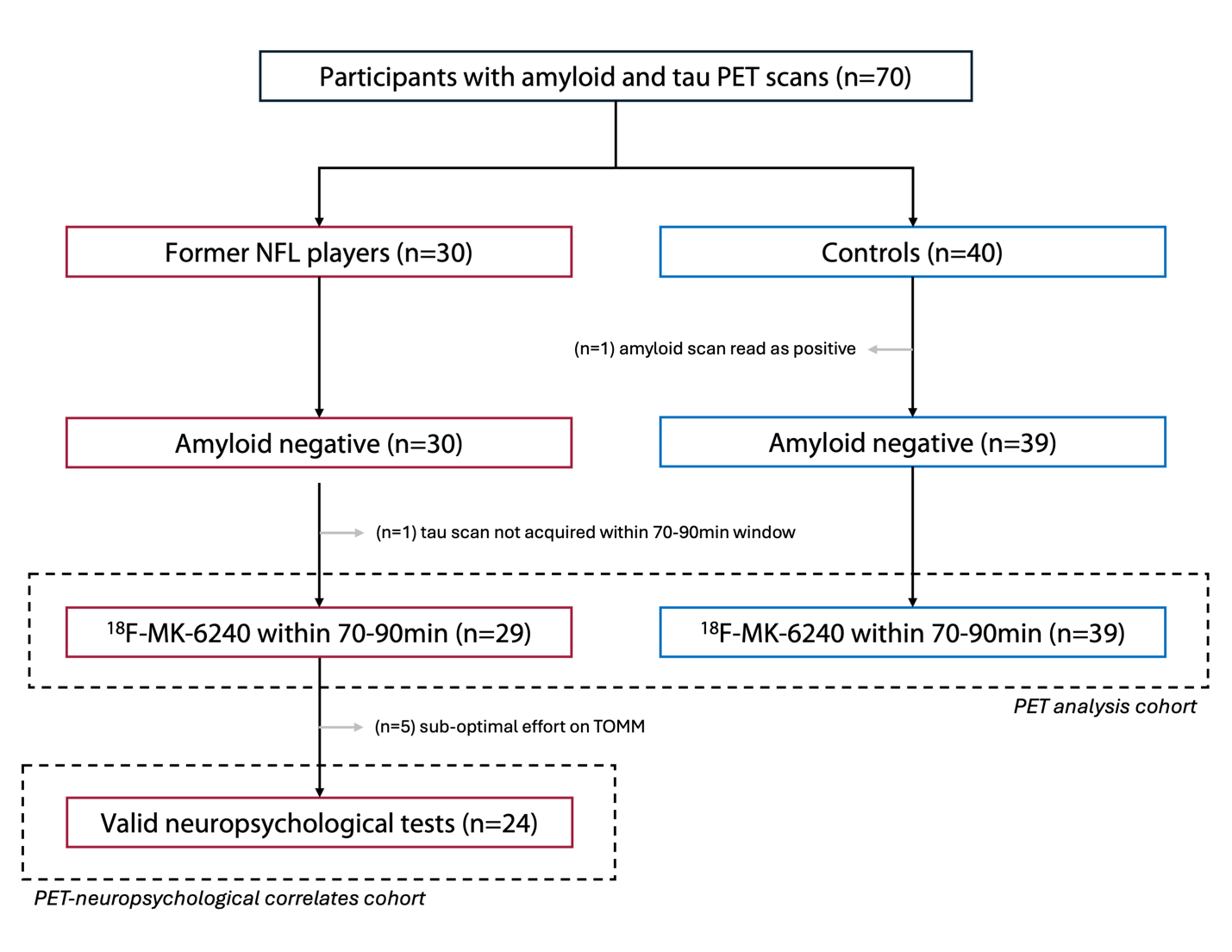


**eFigure 2. ^18^F-MK-6240 SUVr case with visual off-target binding conflicting with cortical binding.** Slices are shown from an ^18^F-MK-6240 scan and coregistered T1-MRI from a single NFL participant (age 45-49). White arrows point to regions with off-target tracer binding. In coronal and sagittal slices, high off-target signal inferior to the temporal lobe is observed bleeding into MTL and basal temporal cortices. In axial slices, smaller pockets of off-target binding are observed in cerebellar gray matter, meninges, dorsal venous sinus, white matter, and skull.


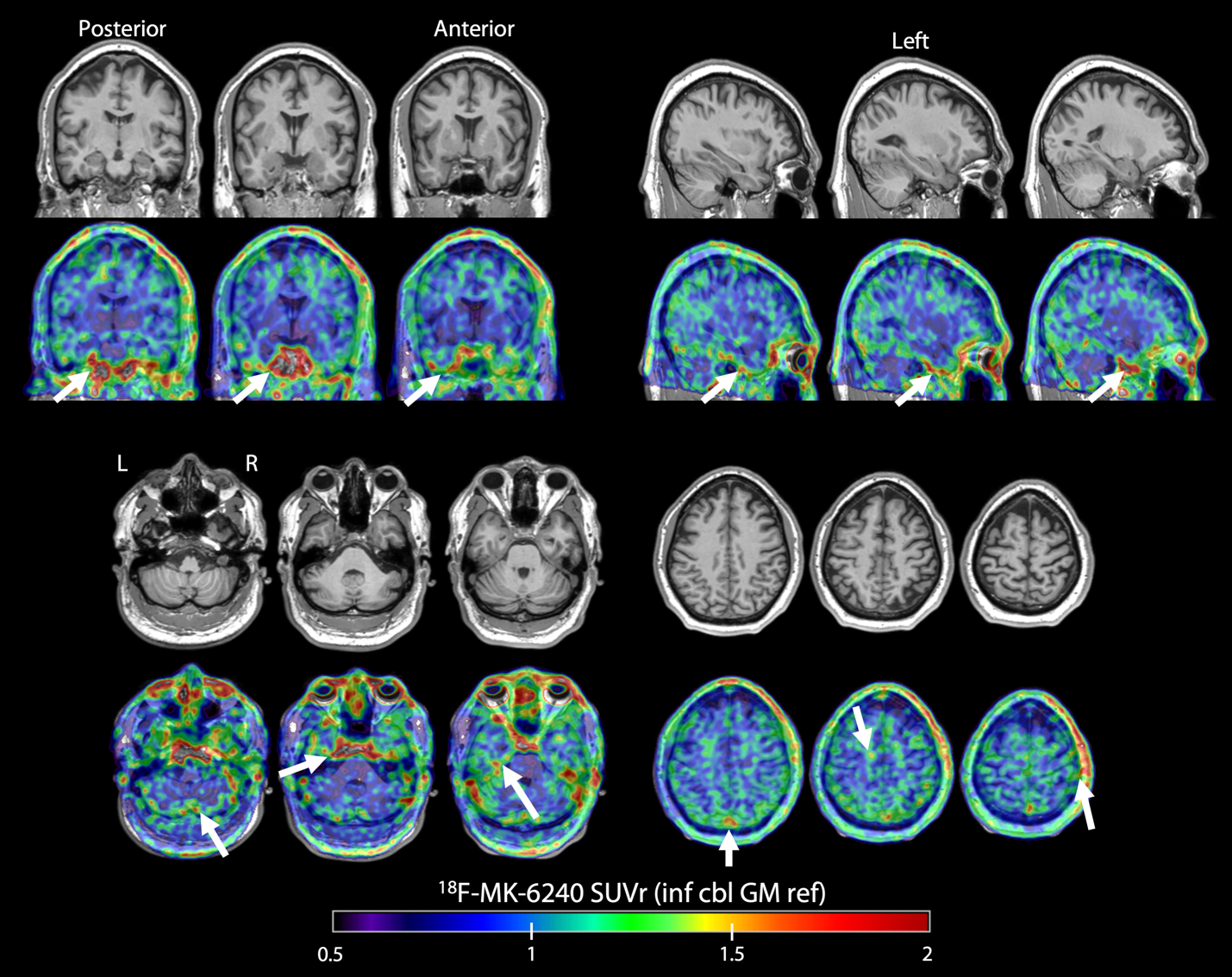


**eFigure 3. ^18^F-MK-6240 SUVr in regions outside the MTL.** Each subplot shows the distribution of mean SUVr values for 29 former NFL players (red points) and 39 cognitively unimpaired controls (blue points) in cortical and subcortical regions-of-interest outside the MTL. *P*-values correspond to values from a one-tailed *t-*test (patients > controls) in a multiple linear regression predicting SUVr as a function of diagnostic group and age. Scatter points from individual participant scans in Figure 2 are labeled if they were outside the first and third quartiles (i.e. outside the box) for each region. For example, label “1” corresponds to Figure 2 NFL 1.


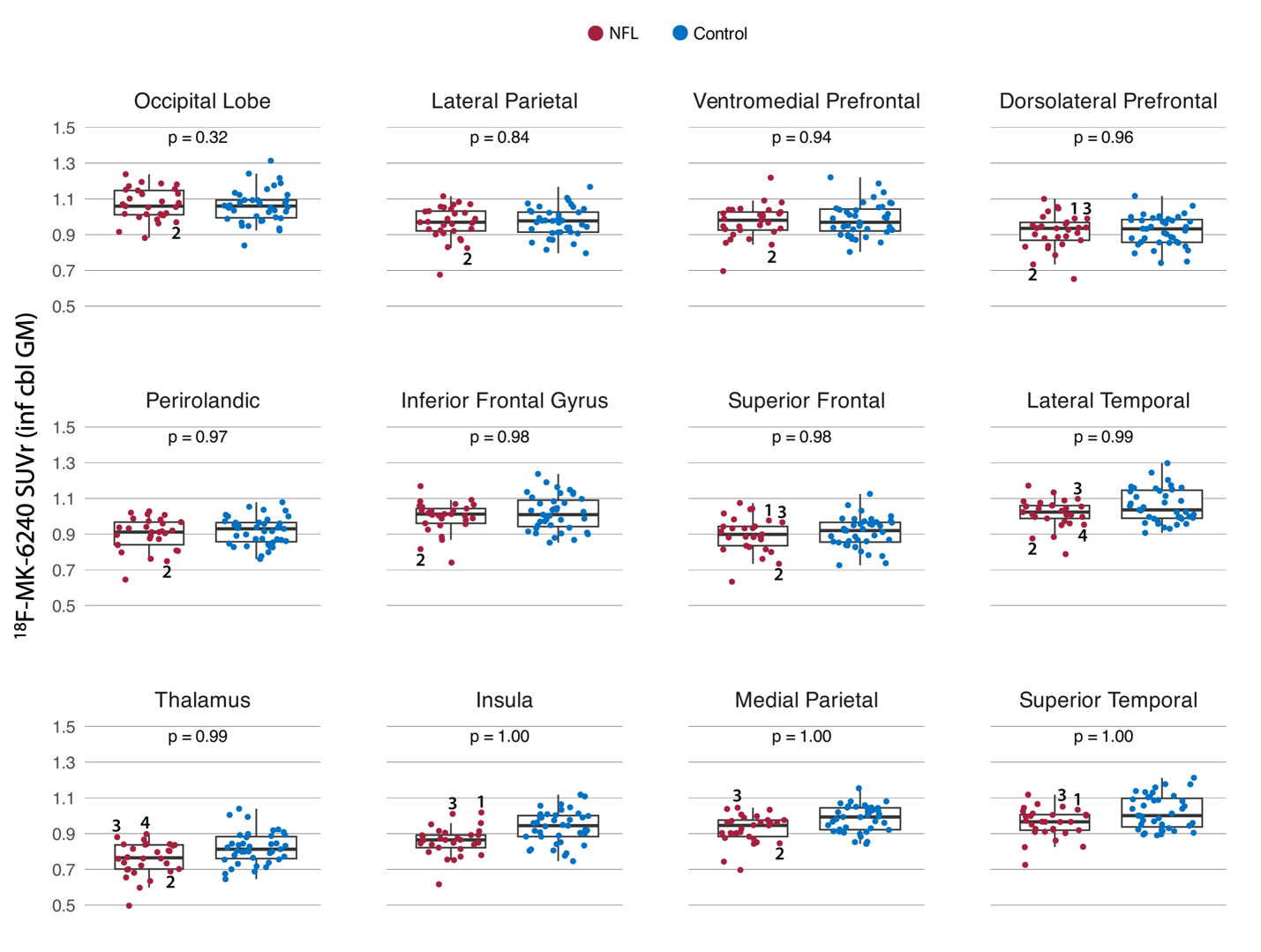

Supplement: Supplementary file 1 — Supplementary Material 1. [file 13024_2025_808_MOESM1_ESM.docx]
